# Tracing the relationship among HIV-1 sub-subtype F1 strains: a phylodynamic perspective

**DOI:** 10.1590/0074-02760220109

**Published:** 2023-01-20

**Authors:** Gabriela Porto Santos Almeida Silva, Rodrigo Cunha Oliveira, Juliana Sacramento Mota de Souza, Marta Giovanetti, Monick Lindenmeyer Guimarães, Carlos Brites, Joana Paixão Monteiro-Cunha

**Affiliations:** 1 Universidade Federal da Bahia, Departamento de Bioquímica e Biofísica, Salvador, BA, Brasil; 2 Universidade Federal de Minas Gerais, Instituto de Ciências Biológicas, Laboratório de Genética Celular e Molecular, Belo Horizonte, MG, Brasil; 3 Fundação Oswaldo Cruz-Fiocruz, Instituto Oswaldo Cruz, Laboratório de AIDS e Imunologia Molecular, Rio de Janeiro, RJ, Brasil; 4 Universidade Federal da Bahia, Faculdade de Medicina, Salvador, BA, Brasil

**Keywords:** HIV, F1 sub-subtype, phylodynamic, recombinants, BF1

## Abstract

**BACKGROUND:**

The human immunodeficiency virus type 1, F1 sub-subtype (HIV-1 F1) circulates in three continents: Africa, Europe, and South America. In Brazil, this sub-subtype co-circulates with subtypes B and C and several recombinant forms, mainly BF1 variants.

**OBJECTIVES:**

This study aimed to reconstruct the dynamic history of HIV-1 F1 in Brazil.

**METHODS:**

HIV-1 near full-length genome and pol gene nucleotide sequences available in public databases were assembled in two datasets (POL671 and NFLG53) to cover the largest number of F1 sub-subtype sequences. Phylodynamic and temporal analyses were performed.

**FINDINGS:**

Two main strains of the F1 sub-subtype are circulating worldwide. The first (F1.I) was found among Brazilian samples (75%) and the second (F1.II) among Romanian (62%) and other European and African isolates. The F1 subtype epidemic in Brazil originated from a single entry into the country around 1970. This ancestral sample is related to samples isolated in European countries (France, Finland, and Belgium), which are possibly of African origin. Moreover, further migration (1998 CI: 1994-2003) of strains from Brazil to Europe (Spain and the UK) was observed. Interestingly, all different recombinant BF patterns found, even those from outside Brazil, present the same F1 lineage (F1.I) as an ancestor, which could be related to the acquisition of adaptive advantages for the recombinant progenies.

**MAIN CONCLUSIONS:**

These findings are important for the understanding of the origin and dynamics of the F1 sub-subtype and a consequent better and greater understanding of the HIV-1 F1 and BF epidemic that still spreads from Brazil to other countries.

The human immunodeficiency virus type 1 (HIV-1) infection is still a major public health problem worldwide with an estimated 38 million people living with HIV by the end of 2020. In the same year, 690,000 people died of HIV-related causes and nearly 1.5 million people were newly infected.^1^ In Brazil, 342,459 new HIV-1 cases were reported in the last 13 years (2007 to June 2020), of which 41,909 occurred in 2019.^2^ The control of viral infection by the host’s immune system and the effectiveness of drugs and vaccines are greatly hampered by the genetic diversity of the virus. The HIV is classified into two types (HIV-1 and HIV-2); HIV-1 is the most prevalent worldwide, further divided into four groups (M, N, O, and P).^3^ The M group has spread significantly, representing around 90% of all infections and diversifying into subtypes, identified as A (sub-subtypes A1, 2, 3, 4, 6 and 7), B, C, D, F (sub-subtypes F1 and F2), G, H, J, K, and L.^4^ ^,^ ^5^ Additionally, different strains can recombine generating intersubtype forms that can be classified as Unique Recombinants Forms (URFs) or Circulating Recombinant Forms (CRFs) when identified by near full-length genome (NFLG) sequencing in at least three non-epidemiologically related individuals.^6^ Currently, the Los Alamos National Laboratory (LANL) lists 121 HIV-1 CRFs (https://www.hiv.lanl.gov/content/sequence/HIV/CRFs/CRFs.html).

The different genetic variants of HIV have a heterogeneous distribution around the world. A recent systematic review estimated a global prevalence of 46.6% for subtype C, 12.1% for subtype B, 10.3% for subtype A, and 0.9% for subtypes F, H, J, and K. In the same study, URFs and CRFs corresponded to approximately 22.8% of HIV infections globally from 2010 to 2015^7^, and 9.6% of these were caused by URFs described in Latin America.^4^

The HIV-1 F1 was initially described by Dumitrescu et al.^8^ in 1994, isolated from Romanian individuals. In Brazil, the first identification of the F1 strain occurred in four samples collected between 1989-1990, the same period that the virus was identified in Romania.^9^ ^,^ ^10^ Later, the analysis of African sequences of the F subtype allowed its classification into two sub-subtypes: F1 and F2.^11^ Despite its low global prevalence, sub-subtype F1 is widely spread, being exceptionally prevalent in some specific countries of Central Africa, South America, and Europe.^12^ Molecular epidemiology studies of HIV-1 F1 in Europe have been conducted, especially in Romania, where this subtype is epidemic and high prevalence rates were found among children and adults.^13^ In South America, the first F1 sequences were described in Brazil^9^ ^,^ ^10^ and Argentina^14^, through partial analysis of the envelope region. In the subsequent years, many partial genomic sequences of the F1 sub-subtype were collected from infected individuals in those two countries, but, after the analysis of larger genomic regions, most of the sequences were confirmed as BF1 recombinant viruses instead of pure F1 strains.^15^ In fact, viruses with unique BF recombination patterns (URF) circulate in Brazil and more than half of the 19 BF circulating recombinant forms (CRF_BF) were first identified in this country.

Phylogenetic studies allow the improvement of the understanding of viral history. In the present study, we used nucleotide sequences from worldwide to reconstruct the evolutionary movement of HIV-1 F1 in Brazil and other countries. This work gathers information on the biological diversity and genetic classification of sub-subtype HIV-1 F1 in Brazil. The generated data provide significant information for understanding the origin and dynamics of HIV-1 F1, which can be useful for the development of measures to control its dissemination.

## MATERIALS AND METHODS

*Dataset* - To understand the transmission dynamics of the HIV-1 F1 sub-subtype in Brazil, nucleotide sequences of *pol* region and NFLG were retrieved from the HIV LANL (http://www.hiv.lanl.gov) and NCBI (http://www.ncbi.nlm.nih.gov) databases. All sequences available in the databases classified as F1 in the NFLG and *pol-protease/reverse transcriptase (pol-PR/RT)* region were collected. The *pol* fragment was chosen because it has the largest number of available sequences, due to its use in monitoring HIV drug resistance. Exclusion criteria of samples included the presence of recombination in the analysed region, presence of gaps, degenerate bases, undefined fragments, stop codons, missing information about sample collection year, sample location, and/or sequences with any major HIV-1 drug resistance mutations according to the Stanford database (https://hivdb.stanford.edu/, updated Oct 23, 2020). All collected sequences were renamed with a code of sample information (access number + country + city + year). From that, two datasets were built. The first dataset included 671 genome sequences (POL671) covering a *pol* region corresponding to positions 2367-3161 (relative to HXB2 reference strain). Out of these, 662 were obtained from public databases and nine were obtained in the state of Bahia, northeastern Brazil through another ongoing study of our group: 4 F1 sequences (006, 015, 192, 203) and 5 BF1 sequences (059, 069, 109, 189, 200), which were classified as F1 throughout the analysed genomic fragment. Among the 662 previously identified sequences, 632 were pure F1 in the entire genome and 30 had fragments of subtype B or BF1 over other genomic regions. The POL671 dataset included sequences from Brazil and other 36 countries [Table and Supplementary data (Table I)], and the second dataset (NFLG53) comprised 53 NFLG sequences (positions790-8898 relative to HXB2) from Brazil and other 12 countries [Table and Supplementary data (Table II)]. The NFLG sequences were chosen as they have the largest genomic representation, even with relatively fewer available sequences.

**TABLE:** Origin of HIV-1 sub-subtype F1 isolates

Country	POL671(N)	NFLG53 (N)
Brazil	281	24
Romania	184	2
Spain	61	13
Italy	20	
Angola	19	3
Portugal	12	
Germany	9	1
Cape Green	8	
CD	8	
United States	7	
Austria	6	
Japan	6	
United Kingdom	6	3
Argentina	5	1
Belgium	5	1
Cyprus	5	1
China	4	
France	3	2
Turkey	3	
French Guyana	2	
Russian Federation	2	1
Bolivia	1	
Bulgaria	1	
Cameroon	1	
Canada	1	
CF	1	
Cuba	1	
Finland	1	1
Luxembourg	1	
Netherlands	1	
Nigeria	1	
Panama	1	
Poland	1	
Slovenia	1	
Sweden	1	
Zimbabwe	1	

N: number of sequences; CD: Democratic Republic of Congo; CF: Central African Republic.

*Sequence alignment and phylogenetic analysis* - Alignment was performed using the MAFFT online program (http://mafft.cbrc.jp)^16^ under the command: mafft-thread 8 - threadtb 5 -threadit 0 -reorder -auto input> output and manually edited using the BioEdit software.^17^ Datasets were assessed for the presence of phylogenetic signals by applying the likelihood mapping analysis implemented in the TreePuzzle Program.^18^ The percentage of fully resolved probability mapping quartets totals was 93.7% for pol dataset and 95.1% for NFLG dataset proving the phylogenetic signal for both tree reconstructions. The maximum likelihood (ML) trees were reconstructed using IQ-TREE 1.6.8 webserver.^19^ Phylogenetic analyses were performed under the GTR+I+G nucleotide substitution model calculated in Modelfinder.^20^ The reliability of each cluster was evaluated considering a bootstrap (BS) of 1,000 replicates and with an approximate likelihood-ratio test (aLRT) based on the Shimodaira-Hasegawa-like procedure.^21^ The ML trees were visualised with FigTree program version 1.4.4 (http://tree.bio.ed.ac.uk/software/figtree/). The subtype was confirmed through phylogeny using the ML method against 11 HIV-1 reference sequences (A1, B, C, D, F2, G, and K) obtained from Los Alamos (https://www.hiv.lanl.gov/content/sequence/NEWALIGN/align.html) and through the Hidden Markov Model method (jpHMM) to exclude possible events of recombination in the samples within the analysed fragment.^22^

*Phylodynamic and temporal analysis of HIV-1 subtype F1* - For phylodynamic studies, ML trees were used to regress root-to-tip genetic distances against sample collection dates using TempEst v 1.5.1 (POL r^2^ = 0.5071; NFLG r^2^ = 0.6337).^23^ In both datasets (NFLG53 and POL671), the BEAST package software 1.10.4 was applied to verify the marginal likelihood values by estimating Path sampling and Sample Stepping-stone to define the model and molecular clock.^24^ ^,^ ^25^ Through the standard nucleotide substitution model (HKY) and the uncorrelated relaxed clock and exponential model, the maximum clade credibility trees (MCC) were obtained. The ML phylogeny was used as a starting tree for Bayesian time-scaled phylogenetic reconstruction. In the analysis of the *pol* fragment, four runs of Markov Chain Monte Carlo (MCMC) with 300 million states each were computed, sampling every 30 million steps, that value of effective sample size (ESS) of proper mixing was 334.2 (> 200). The log and trees files were combined in LogCombiner discarding 10% in each run (POL671 = 40%) and (NFLG53 = 20%) as burn-in; the convergence of the MCMC chains was verified using Tracer v.1.7.1.^26^ The Bayesian coalescent inference of the population analysis was performed with the NFLG dataset based on the MCMC model and chain number already described.

## RESULTS

*Dataset characterisation* - The POL671 dataset comprised 39 sequences from Africa, 289 from South America, 11 from Central/North America, 17 from Asia, and 316 from Europe. The oldest sequence was obtained in 1989 and the most recent was collected in 2019. Regarding the NFLG53 dataset, among the 53 sequences, one was collected in Asia, three in Africa, 35 in South America, and 24 in Europe. The oldest sequence was collected in 1989 and the most recent in 2016.

**Fig. 1:**
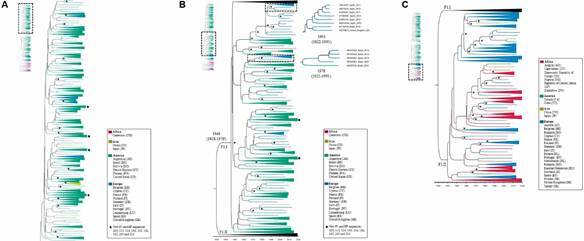
time scale Bayesian maximum clade credibility trees (MCC) (n = 671) based on pol genomic fragment. Tree was reconstructed using 671 HIV-1 sub-subtype F1 viral isolates from worldwide. (A) with emphasis on the tree top (B) middle and (C) bottom. Sequences were isolated in Brazil (280), Europe (316) and Africa (39). Branches with PP support (> 0.80) are marked with a *. The colours of the branches refer to the location indicated by the legend. New Brazilian sequences are marked with ♦. Relevant branches were expanded for better visualisation, exposing its dating and confidence interval. The cluster of Brazilian sequences is shown in Fig. 1A-B (F1.I lineage) and collapsed in 1C for better visualisation of the lower branch of the tree (F1.II lineage).

*Phylogenetic inference* - The ML analysis of POL671 [Supplementary data (Fig. 1)] dataset shows a topology with two large nodes. The F1.I lineage supported by aLRT = 85/ BS = 92 includes sequences from 20 countries (Brazil, Spain, Germany, Portugal, Luxembourg, Italy, Argentina, Bolivia, United States, Japan, French Guyana, China, France, Finland, Belgium, United Kingdom, Germany, Cameroon, Cyprus, and Panama). Most of the samples in this upper cluster belong to South America, including all sequences from Brazil (n = 281). All 35 F1 sequences retrieved from BF1 recombinants sequences, regardless of origin, clustered inside the clade F1.I. Furthermore, only one African F1 sample (from the Republic of Cameroon aLRT 85/BS 99) was inside this group. The F1.II lineage grouped samples from 25 countries (Romania, China, Japan, Belgium, Spain, Canada, Angola, Portugal, Germany, Cape Green, Nigeria, Democratic Republic of Congo, Russian Federation, France, Central African Republic, Cuba, Slovenia, Sweden, Italy, United Kingdom, Netherlands, Cyprus, Turkey, Poland, and Austria) with aLRT = 85/BS = 82. Notably, most sequences from African countries (97.4%) were in this cluster. The external group of this cluster is represented by five sequences from African countries (Angola, Democratic Republic of Congo, and Zimbabwe [aLRT 84/BS 99].

**Fig. 2:**
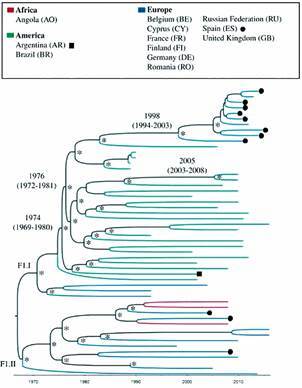
time scale Bayesian maximum clade credibility trees (MCC) (n = 53) based on near full-length genome (NFLG) sequences showing the phylogenetic relationships among sub-subtype F1 viruses circulating in Brazil (25), Europe (24) and Africa (3). Branch colors represents the geographic region of origin, according to the legend. Important branches with PP support (> 0.80) are marked with *. Horizontal branch lengths are drawn to scale with the bar at the bottom indicating years. F1.I represents the cluster of Brazilian sequences.

Of note, in the NLFG analysis, the two main nodes were also present in the ML tree [Supplementary data (Fig. 2)] (F1.II node: aLRT 99/ BS 100 and F1.I: aLRT 99 / BS 100), confirming the separation between the strains circulating in Brazil from those found in Romania and Africa. The ancestor of the F1.I node seems to originate from two clusters: one with Brazilian sequences and a second cluster with European sequences only (from Finland, France, and Belgium).

To understand the transmission dynamics of HIV-1 F1 in Brazil, Bayesian analyses were conducted, and the topologies obtained from both datasets (Figs 1-2) were remarkably similar to the ML trees [Supplementary data (Figs 1-2)], showing the two major F1 lineages circulating worldwide. Again, Brazilian, and Romanian sequences were always grouped in distinct clusters. In the Bayesian tree of pol (Fig. 1), the F1.I cluster was composed of 374 sequences, of which 280 (74.5%) came from Brazil. The common ancestor of this cluster would have circulated in 1946 (CI:1928-1959). The F1.II cluster grouped 297 sequences, mostly from Romania (84.6%). All new Brazilian sequences from the state of Bahia were intermixed with previously described Brazilian F1 sequences inside the F1.I clade, with high PP support. As in the ML tree, all 35 BF recombinant sequences are also grouped inside this clade.

The NFLG Bayesian tree (Fig. 2) shows, as in POL671 tree, an F1.II node with European and African sequences and an F1.I node with Brazilian sequences. The estimated time to the most recent common ancestor (TMRCA) of this Brazilian lineage is 1974 (CI: 1969-1980). This reconstruction also points to a subsequent migration of the F1.I lineage from Brazil to Europe (Spain and UK) in the late 1990s (1998 with CI 1994-2003 [PP = 1]).

**Fig. 3:**
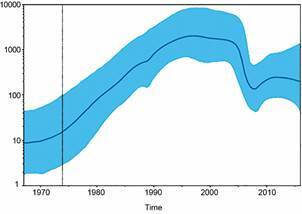
Bayesian coalescent inference of population size of subtype F1 from dataset near full-length genome (NFLG)53 including 53 NFLG sequences (positions: 790-8898 relative to HXB2) from Brazil and others 11 countries (Angola, Argentine, Belgium, Cyprus, France, Finland, Germany, Romania, Russian Federation, Spain and United Kingdom). All sequences were obtained from the Los Alamos HIV sequence database (www.hiv.lanl.gov/content/index).

*Bayesian coalescent inference of population analysis* - The Bayesian coalescent inference of population analysis from the NFLG dataset generated a population growth curve with a time interval between 1970 and 2015 (Fig. 3). This result shows the exponential growth of the F1 subtype until around 1995; from then on, there is a plateau between 1995 and 2005, followed by a decrease in the expansion of this subtype and a slight increase around 2009.

## DISCUSSION

The two different datasets gave rise to ML and Bayesian trees with remarkably similar topologies. In all four trees [Figs 1-2, and Supplementary data (Figs 1-2)], one can observe the existence of a relationship between sequences from South American and European countries (F1.I node), more specifically between Brazil and Spain. Also, in the F1.II there is evidence of the phylogenetic link between samples from African and European countries; in this case, Romania is the most represented country. The F1 lineage in Brazil is more related to sequences from France, Finland, and Belgium, and these are close to F1 African sequences (mainly West and Central Africa).^27^ ^,^ ^28^

Furthermore, in the four analyses, all Brazilian sub-subtype F1 sequences formed a monophyletic group together with sequences from other regions (F1.I large group). This indicates a single entry of sub-subtype F1 in Brazil around the 70s, as shown in our Bayesian analyses (Figs 1-2). Earlier evolutionary analyses revealed that the oldest isolates related to the F1 sequences were from the Democratic Republic of Congo and that their diversification occurred in that country in the late 1950s.^29^ In Brazil, studies on the evolutionary history of the HIV F1 sub-subtype estimated that the onset of the epidemic occurred in the early 1980s.^30^ ^,^ ^31^ ^,^ ^32^ In contrast to these findings, evaluating the tMRCA data from our trees, it is notable that, in the analysis of the pol region, 12 out of 27 clusters involving Brazilian samples with PP ≥ 0.80 were dated around the 1970s, consistent with others.^12^ ^,^ ^15^ In addition, the NFLG53 tree (Fig. 2) shows the probable introduction of HIV-1 F1 in Brazil by 1974 (PP = 0.98). Our findings coincide with the end of Angola’s independence war and the beginning of the civil war in 1975, which was followed by a wave of emigration and the participation of foreign troops from several countries.^27^ ^,^ ^33^

The HIV-1 F1 sub-subtype viral strain has been identified at the same time in Brazil and Romania. For this reason, a premeditated conclusion hypothesises an epidemiological and evolutionary relationship between the F1 viruses from these two countries. Despite of this, in all four phylogenetic trees of this study, the sequences from Romania and Brazil grouped into two distinct clusters. In the Brazilian monophyletic group, there are no sequences from Romania, as they were always grouped in the F1.II unlinked node. Bandea et al.^34^ in their phylogenetic analyses also showed that there is no epidemiological link between the two epidemics; Guimarães et al.^29^ showed that the F1 strains from Romania and Brazil were grouped into two related but distinct clusters. In this context, this research reinforces previous findings with greater grounding and support for phylogenetic analyses.

The European subcluster inside the F1.I cluster in *pol* (Fig. 1B) and NFLG trees (Fig. 2) points to a recent relationship among Brazilian, Spanish, and English viruses. According to these reconstructions (ML and Bayesian), the common ancestor of this European clade originated from the Brazilian cluster and dated from 1998 (CI 1994-2003), suggesting that there may be a contribution of the Brazilian F1 sub-subtype to the HIV-1 F1 epidemic in Europe, mainly Spain. This inference is supported by the recent spread of subtype F to new geographic areas (especially Spain) and its later expansion, showing a high rate of an effective number of infections in a short period.^35^ Additionally, in the last decades, Spain has become one of the main destinations in Europe for immigrants and one of the western European countries with the highest rates of HIV infections.

The *pol* dataset holds 35 BF1 recombinants that were classified as F1 throughout the genomic fragment used in our analyses. All these recombinant sequences, including three non-Brazilian viruses (Japan, Bolivia, and Argentina), were inside the F1.I cluster, together with Brazilian F1 strains in both Bayesian (Fig. 1) and ML [Supplementary data (Fig. 1)] trees. It has been shown that BF1 recombinant sequences have many distinct recombination patterns, indicating different recombination events.^36^ In the present study, despite the different recombination patterns observed among the 35 BF1 representatives, the clustering of all recombinants in the same monophyletic cluster suggests a common origin for these viruses. This may indicate that the pure F1 Brazilian ancestor could have arisen from a possible evolution after adaptive advantages associated with recombinant strains. Our results indicate that all BF1 recombinants in the present study have the same F1 lineage as the ancestral sequence, which could show a tendency of this ancestral lineage to recombine and the existence of adaptative advantages for the recombinant progenies.

The results of this study show a probable migration of the F1 sub-subtype from Africa to Europe and, after that, a single introduction of this strain to Brazil around the 70s (Figs 1A-B, 2). From these analyses, it is also possible to suggest a later migration from South America to Europe in 1998 (1994-2003) (Fig. 2). The consistency of these results is reflected by the greater number of sequences analysed, compared to earlier studies that focused on a single, smaller genomic region, and by the consistency of the topologies observed in *pol* and NFLG datasets in both Bayesian and ML analyses. These findings provide important information, involving molecular epidemiology, which contributes to understanding the origin, diversification, and dispersion dynamics of the F1 sub-subtype. This knowledge contributes to the surveillance and control of the HIV-1 F1 and BF1 epidemic that is still spreading from Brazil to South America and other European countries.

The Bayesian coalescent inference of population growth showed a temporal expansion of the F1 sub-subtype in the first decades of the epidemic, followed by the stabilisation of transmission. Lima et al.^32^ compared a limited number of F1 samples from Pernambuco against F1 reference sequences between 1986 and 2010 and found that both datasets (Pernambuco and reference) were associated with a moderate growth phase followed by a significant increase in the frequency of HIV-1 F strains. Their plot also shows a plateau between 2005 and 2010, which corroborates our analysis, showing a slight decrease in the expansion of the F1 sub-subtype over the same period. Furthermore, Bello et al.^12^ also showed the slow but constant growth of the F1 epidemic in adults, until the beginning of the 2000s; after that, the epidemic growth rate started to slow down. Based on temporal expansion analyses, our study included all HIV-1 F1 NFLG sequences available in the databases up to the year of collection. Despite of this, we suggest that further studies should be conducted to evaluate the expansion of the F1 sub-subtype over the next few years, based on the availability of new sequences. Furthermore, we believe that new temporal expansion analyses can be effective for a comparison between recombinant and pure F1 to achieve further interpretations of adaptive advantages.
