# Supplementary material for: Tracing the relationship among HIV-1 sub-subtype F1 strains: a phylodynamic perspective
Source: Mem Inst Oswaldo Cruz. 2023 Jan 20;117:e220109. doi: 10.1590/0074-02760220109 (PMC9870255; doi:10.1590/0074-02760220109)
Supplement: Supplementary file 1 [file 1678-8060-mioc-117-e220109-s.pdf]

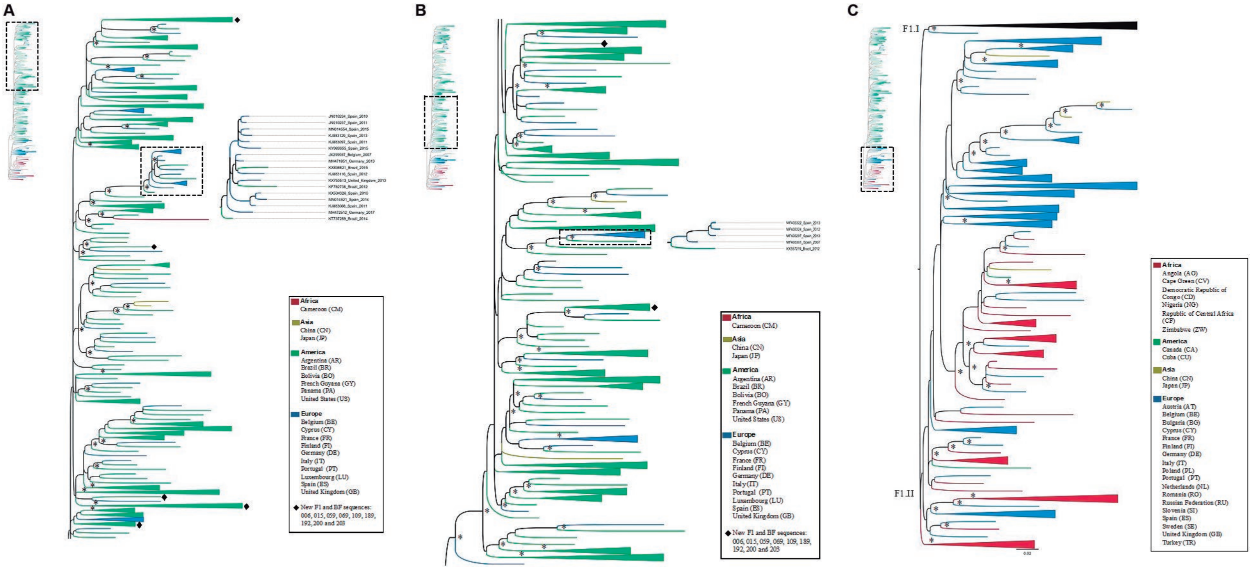

Fig. 1: maximum likelihood tree based on pol genomic region showing the phylogenetic relationships of 671 HIV-1 subtype F1 viral isolates from worldwide. (A) tree top, (B) middle of the tree and (C) tree base. The sequences were isolated in Brazil (n = 280), Europe (n = 316) and Africa (n = 39). Important branches with Bootstrap (BT) support (> 90) are marked with a (*). The branch color represents the location indicated by the legend. Recombinant sequences are marked with (♦). Relevant branches were expanded for better visualisation. The cluster containing Brazilian sequences is shown in 1A and 1B (called F1.I) and collapsed in 1C.

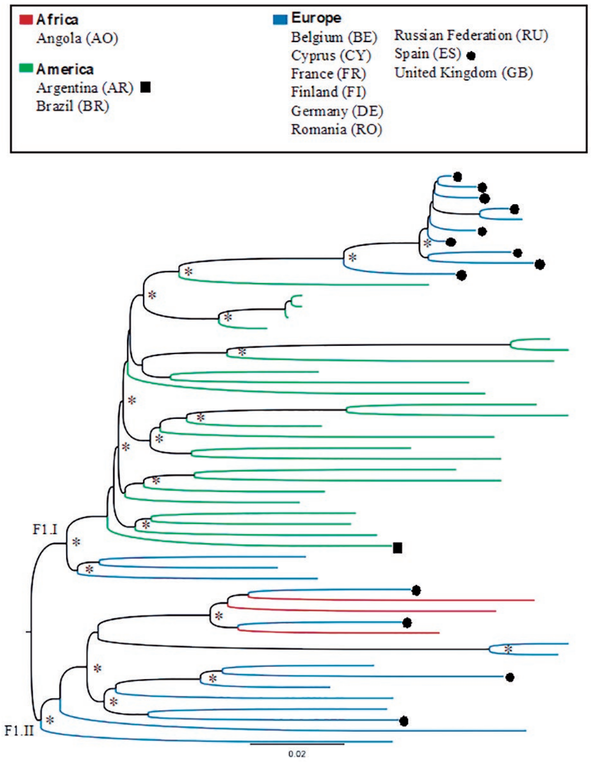

Fig. 2: maximum likelihood tree (n = 53) based on near-full length genome (NFLG) sequences showing the phylogenetic relationships among subtype F1 viruses circulating in Brazil (25), Europe (24) and Africa (3). The branch colour represents the geographic region from where the subtype F1 strain originated, according to the legend given in the figure. Important branches with Bootstrap (BT) support (> 90) are marked with a (*). Brazilian sequences are clustered inside the F1.I branch.

cc BY

TABLE I
Identification of the analysed sequences of POL671 dataset

Source subtype	Accession number or id	Country	Sample year
F1	006	Brazil	2017
F1	015	Brazil	2018
BF1	059	Brazil	2018
BF1	069	Brazil	2018
BF1	109	Brazil	2019
BF1	189	Brazil	2019
F1	192	Brazil	2019
BF1	200	Brazil	2019
F1	203	Brazil	2019
F1	AB485657	Brazil	1990
F1	AB640464	Japan	2003
F1	AB864437	Japan	2012
F1	AB866092	Japan	2009
BF1	AB866302	Japan	2011
F1	AB866775	Japan	2008
F1	AF005494	Brazil	1993
F1	AF075703	Finland	1993
F1	AF077336	Belgium	1993
F1	AF112897	Brazil	1997
F1	AF112909	Brazil	1997
F1	AF204039	Romania	1993
F1	AF204040	Romania	1997
F1	AF204041	Romania	1993
F1	AF204042	Romania	1993
F1	AF204043	Romania	1996
F1	AF204044	Romania	1996
F1	AF204045	Romania	1996
F1	AF204046	Romania	1997
F1	AF204047	Romania	1997
F1	AF204049	Romania	1997
F1	AF204050	Romania	1997
F1	AF204051	Romania	1997
F1	AF204052	Romania	1997
F1	AF295281	Italy	1997
F1	AF295282	Italy	1998
F1	AF295283	Italy	1998
F1	AF295299	Italy	1998
BF1	AF447815	Brazil	1990
F1	AF447826	Brazil	1989
F1	AF447853	Romania	1994
F1	AF447857	Romania	1994
F1	AJ287042	France	1999
F1	AJ971100	Slovenia	2002

Source subtype	Accession number or id	Country	Sample year
F1	AM041018	Democratic Republic of Congo	2002
F1	AM041021	Democratic Republic of Congo	2002
F1	AY275717	Brazil	2002
F1	AY275729	Brazil	2002
F1	AY359521	Italy	2000
F1	AY999358	Brazil	1998
F1	AY999378	Brazil	1998
F1	AY999386	Brazil	1998
F1	AY999388	Brazil	1998
F1	AY999427	Brazil	1998
F1	AY999449	Brazil	1998
F1	AY999463	Brazil	1998
F1	AY999481	Brazil	1998
F1	AY999511	Brazil	1998
F1	AY999530	Brazil	1998
F1	AY999531	Brazil	1998
F1	AY999532	Brazil	1998
F1	AY999541	Brazil	1998
F1	AY999569	Brazil	1998
F1	AY999603	Brazil	1998
F1	AY999673	Brazil	1998
F1	AY999679	Brazil	1998
F1	DQ156240	Brazil	2006
F1	DQ156249	Brazil	2006
F1	DQ156261	Brazil	2006
F1	DQ156270	Brazil	2006
F1	DQ156277	Brazil	2006
F1	DQ156281	Brazil	2006
F1	DQ156287	Brazil	2006
F1	DQ189088	Argentina	2002
F1	DQ518444	Brazil	2004
F1	DQ899709	Brazil	2004
F1	EF042646	Brazil	2004
F1	EF042648	Brazil	2005
F1	EF042650	Brazil	2005
F1	EF042667	Brazil	2005
F1	EF042688	Brazil	2006
BF1	EF120104	Argentina	2003
F1	EF379155	Brazil	2004
F1	EF379163	Brazil	2005
F1	EF379175	Brazil	2005
F1	EF379184	Brazil	2006
F1	EF379192	Brazil	2006
F1	EF379197	Brazil	2006
F1	EU189701	Brazil	2006

Source subtype	Accession number or id	Country	Sample year
F1	EU189702	Brazil	2005
F1	EU248476	Belgium	2003
F1	EU340743	Brazil	2006
F1	EU741668	Italy	2006
F1	EU822584	Spain	2005
F1	EU822585	Spain	2005
F1	EU822710	Spain	2007
F1	EU822712	Spain	2007
F1	EU822753	Spain	2007
F1	FJ009814	Russian Federation	2006
F1	FJ030690	Italy	1998
F1	FJ405143	Brazil	2002
F1	FJ405145	Brazil	2003
F1	FJ405149	Brazil	2003
F1	FJ405152	Brazil	2002
F1	FJ405168	Brazil	2002
F1	FJ405178	Brazil	2002
F1	FJ481659	Spain	2007
F1	FJ481660	Spain	2004
F1	FJ548805	Brazil	2007
F1	FJ591245	Brazil	2004
F1	FJ591608	Brazil	2004
F1	FJ591612	Brazil	2004
F1	FJ594169	Brazil	2007
F1	FJ594180	Brazil	2007
F1	FJ594185	Brazil	2007
F1	FJ670516	Spain	2002
F1	FJ771009	Brazil	2006
F1	FJ784166	Brazil	2003
F1	FJ784189	Brazil	2003
F1	FJ784194	Brazil	2003
F1	FJ784209	Brazil	2003
F1	FJ784210	Brazil	2003
F1	FJ784217	Brazil	2003
F1	FJ784224	Brazil	2003
F1	FJ900266	Angola	2006
F1	FJ900267	Angola	2006
F1	FJ900268	Angola	2006
F1	FJ966988	Brazil	2002
F1	FJ967103	Brazil	2004
F1	FJ967107	Brazil	2004
F1	FJ967111	Brazil	2005
F1	GQ241027	Spain	2007
F1	GQ294600	Brazil	2006
F1	GQ398854	Italy	2005

Source subtype	Accession number or id	Country	Sample year
F1	GQ398928	Italy	2003
F1	GQ398932	Italy	2005
F1	GQ398973	Portugal	2005
F1	GQ399041	Austria	2004
F1	GQ399125	Italy	2004
F1	GQ399853	Italy	2004
F1	GQ399904	Portugal	2003
F1	GQ399922	Portugal	2003
F1	GQ399927	Italy	2005
F1	GQ400079	Italy	2004
F1	GQ400186	Austria	2004
F1	GQ400222	Italy	2004
F1	GQ400229	Netherlands	2003
F1	GQ400234	Austria	2004
F1	GQ400408	Italy	2004
F1	GQ400485	Portugal	2004
F1	GQ400958	Sweden	2005
F1	GQ401313	Brazil	2007
F1	GQ862306	Brazil	2007
F1	GQ862307	Brazil	2006
F1	GQ862308	Brazil	2004
F1	GQ862318	Brazil	2007
F1	GQ862323	Brazil	2007
F1	GQ862327	Brazil	2007
F1	GU121170	Brazil	2007
F1	GU288711	Brazil	2004
F1	GU288713	Brazil	2005
F1	GU288728	Brazil	2005
F1	GU288754	Brazil	2005
F1	GU288768	Brazil	2006
F1	GU288801	Brazil	2003
F1	GU326163	Spain	2009
F1	HM024981	Brazil	2005
F1	HM102342	Portugal	2003
F1	HM102343	Portugal	2003
F1	HM102344	Portugal	2003
F1	HM102346	Portugal	2003
F1	HM102347	Portugal	2004
F1	HM102348	Portugal	2004
F1	HM102349	Portugal	2007
F1	HM191558	Romania	2003
F1	HM191560	Romania	2003
F1	HM191561	Romania	2004
F1	HM191562	Romania	2004
F1	HM191563	Romania	2004

Source subtype	Accession number or id	Country	Sample year
F1	HM191564	Romania	2003
F1	HM191565	Romania	2003
F1	HM191568	Romania	2004
F1	HM191569	Romania	2004
F1	HM191570	Romania	2004
F1	HM191571	Romania	2004
F1	HM191572	Romania	2004
F1	HM191573	Romania	2004
F1	HM191574	Romania	2004
F1	HM191575	Romania	2004
F1	HM191576	Romania	2004
F1	HM191577	Romania	2003
F1	HM533996	Brazil	2008
F1	HM534040	Brazil	2009
F1	HM534056	Brazil	2009
F1	HM534075	Brazil	2009
F1	HM534109	Brazil	2009
F1	HM534136	Brazil	2009
F1	HM534137	Brazil	2009
F1	HM534167	Brazil	2009
F1	HM534177	Brazil	2009
F1	HM534188	Brazil	2009
F1	HM583578	Brazil	2008
F1	HQ127493	Brazil	2009
F1	HQ127501	Brazil	2009
F1	HQ127504	Brazil	2009
F1	HQ127552	Brazil	2009
F1	HQ638716	Brazil	2008
F1	HQ638751	Brazil	2009
F1	HQ638774	Brazil	2009
F1	HQ638791	Brazil	2009
F1	HQ667668	Italy	2009
F1	HQ667694	Italy	2009
F1	JF342287	Brazil	2008
BF1	JF342291	Brazil	2008
F1	JF342306	Brazil	2008
F1	JF487884	Brazil	2006
F1	JF683771	Cyprus	2008
F1	JF774010	Brazil	2008
F1	JF929088	Spain	2007
F1	JF929111	Spain	2005
F1	JF929165	Spain	2007
F1	JN010234	Spain	2010
F1	JN010237	Spain	2011
F1	JN010484	Brazil	2005

Source subtype	Accession number or id	Country	Sample year
BF1	JN010604	Brazil	2005
F1	JN010732	Brazil	2005
F1	JN010735	Brazil	2005
BF1	JN029714	Brazil	2006
BF1	JN029747	Brazil	2006
BF1	JN114131	Brazil	2008
BF1	JN114142	Brazil	2009
F1	JN114157	Brazil	2009
F1	JN114167	Brazil	2009
BF1	JN114182	Brazil	2009
BF1	JN114208	Brazil	2010
F1	JN114212	Brazil	2010
F1	JN195832	Brazil	2003
F1	JN195934	Brazil	2007
F1	JN195935	Brazil	2007
F1	JN196017	Brazil	2010
F1	JN196018	Brazil	2010
F1	JN634402	Brazil	2010
F1	JN662426	Brazil	2009
F1	JN670311	Argentina	2005
F1	JN671057	Argentina	2006
F1	JN671097	Argentina	2006
BF1	JN710763	Bolivia	1996
F1	JN937019	Angola	2008
F1	JN937044	Angola	2009
F1	JN937064	Angola	2009
F1	JN937068	Angola	2010
F1	JN937080	Angola	2010
F1	JN937089	Angola	2010
F1	JN937111	Angola	2010
F1	JN937113	Angola	2010
F1	JN937114	Angola	2010
F1	JQ082990	Romania	2003
F1	JQ083004	Romania	2004
F1	JQ083007	Romania	2004
F1	JQ083011	Romania	2004
F1	JQ083012	Romania	2004
F1	JQ083018	Romania	2008
F1	JQ083019	Romania	2007
F1	JQ083024	Romania	2007
F1	JQ083026	Romania	2008
F1	JQ083027	Romania	2008
F1	JQ083035	Romania	2007
F1	JQ083036	Romania	2007
F1	JQ083037	Romania	2007

Source subtype	Accession number or id	Country	Sample year
F1	JQ083039	Romania	2007
F1	JQ083041	Romania	2008
F1	JQ083043	Romania	2008
F1	JQ083046	Romania	2007
F1	JQ083049	Romania	2007
F1	JQ083052	Romania	2007
F1	JQ083053	Romania	2007
F1	JQ083054	Romania	2007
F1	JQ083056	Romania	2007
F1	JQ083059	Romania	2007
F1	JQ083065	Romania	2007
F1	JQ083068	Romania	2007
F1	JQ083076	Romania	2007
F1	JQ280889	Romania	2006
F1	JQ280895	Romania	2006
F1	JQ280896	Romania	2006
F1	JQ280897	Romania	2006
F1	JQ280899	Romania	2006
F1	JQ280900	Romania	2006
F1	JQ280901	Romania	2006
F1	JQ280905	Romania	2007
F1	JQ280907	Romania	2007
F1	JQ280912	Romania	2008
F1	JQ280915	Romania	2008
F1	JQ280917	Romania	2008
F1	JQ280922	Romania	2002
F1	JQ280923	Romania	2003
F1	JQ280924	Romania	2002
F1	JQ280925	Romania	2003
F1	JQ280930	Romania	2003
F1	JQ280933	Romania	2003
F1	JQ280934	Romania	2003
F1	JQ280935	Romania	2003
F1	JQ280936	Romania	2003
F1	JQ280937	Romania	2003
F1	JQ280938	Romania	2003
F1	JQ280939	Romania	2003
F1	JQ280940	Austria	2003
F1	JQ280941	Germany	2003
F1	JQ280942	France	2005
F1	JQ616895	Angola	2009
F1	JQ616904	Angola	2009
F1	JQ619550	Brazil	1998
F1	JQ619570	Brazil	1998
F1	JX299555	Romania	2007

Source subtype	Accession number or id	Country	Sample year
F1	JX299614	Romania	2007
F1	JX299627	Luxembourg	2006
F1	JX299652	Romania	2007
F1	JX299689	Romania	2007
F1	JX299694	Romania	2007
F1	JX299734	Romania	2007
F1	JX299747	Romania	2007
F1	JX299793	Romania	2007
F1	JX299834	Romania	2007
F1	JX299872	Romania	2007
F1	JX299896	Romania	2007
F1	JX299937	Belgium	2007
F1	JX299981	Romania	2007
F1	JX300011	Romania	2007
F1	JX300019	Romania	2007
F1	JX300038	Romania	2007
F1	JX300123	Romania	2007
F1	JX300203	Austria	2007
F1	JX300205	Austria	2007
F1	JX300244	Romania	2007
F1	JX300260	Romania	2007
F1	JX300318	Romania	2007
F1	JX300462	Romania	2007
F1	JX300498	Romania	2007
F1	JX300566	Cyprus	2007
F1	JX300626	Romania	2007
F1	JX300667	Romania	2007
F1	JX300736	Romania	2007
F1	JX300753	Romania	2007
F1	JX300760	Romania	2007
F1	JX300761	Romania	2007
F1	JX300779	Romania	2007
F1	JX300780	Romania	2007
F1	JX300855	Romania	2007
F1	JX300924	Romania	2007
F1	JX300955	Romania	2007
F1	JX301011	Cyprus	2007
F1	JX301047	Romania	2007
F1	JX301048	Romania	2007
F1	JX301153	Romania	2007
F1	JX460243	United States	2009
F1	JX460475	United States	2010
F1	JX460496	United States	2010
F1	JX460497	United States	2010
F1	KC249749	Brazil	2010

Source subtype	Accession number or id	Country	Sample year
F1	KC340132	Spain	2007
F1	KC340133	Spain	2008
F1	KC340378	Spain	2008
F1	KC340396	Spain	2009
F1	KC340440	Spain	2009
F1	KC340499	Spain	2009
F1	KC340853	Spain	2010
F1	KC352099	China	2007
F1	KF701009	Brazil	2012
F1	KF702324	Panama	2012
F1	KF727755	Canada	2007
F1	KF782688	Brazil	2012
F1	KF782738	Brazil	2012
F1	KF921999	Brazil	2005
F1	KF922002	Brazil	2005
F1	KF922009	Brazil	2005
F1	KF922012	Brazil	2005
F1	KF922034	Brazil	2005
F1	KF922041	Brazil	2005
F1	KF922042	Brazil	2005
F1	KF922088	Brazil	2005
F1	KF922097	Brazil	2005
F1	KF922111	Brazil	2005
F1	KF922127	Brazil	2006
F1	KF922129	Brazil	2006
F1	KF922131	Brazil	2006
F1	KF922171	Brazil	2007
F1	KF922183	Brazil	2007
F1	KF922196	Brazil	2007
F1	KF922201	Brazil	2005
F1	KJ194674	Romania	2012
F1	KJ194675	Romania	2012
F1	KJ194679	Romania	2012
F1	KJ194681	Romania	2012
F1	KJ194682	Romania	2012
F1	KJ194684	Romania	2012
F1	KJ194685	Romania	2012
F1	KJ194689	Romania	2011
F1	KJ194693	Romania	2012
F1	KJ194696	Romania	2011
F1	KJ194697	Romania	2011
F1	KJ194698	Romania	2012
F1	KJ194700	Romania	2011
F1	KJ194701	Romania	2011
F1	KJ194703	Romania	2011

Source subtype	Accession number or id	Country	Sample year
F1	KJ194704	Romania	2012
F1	KJ194706	Romania	2012
F1	KJ194707	Romania	2011
F1	KJ194708	Romania	2011
F1	KJ194709	Romania	2011
F1	KJ194710	Romania	2012
F1	KJ194712	Romania	2012
F1	KJ194713	Romania	2012
F1	KJ194715	Romania	2011
F1	KJ194716	Romania	2011
F1	KJ194718	Romania	2012
F1	KJ194735	Romania	2012
F1	KJ194736	Romania	2012
F1	KJ194739	Romania	2012
F1	KJ194742	Romania	2012
F1	KJ194748	Romania	2012
F1	KJ194752	Romania	2012
F1	KJ194756	Romania	2012
F1	KJ194757	Romania	2012
F1	KJ194758	Romania	2012
F1	KJ194760	Romania	2012
F1	KJ194763	Romania	2011
F1	KJ194772	Romania	2012
F1	KJ194775	Romania	2012
F1	KJ194778	Romania	2012
F1	KJ194782	Romania	2011
F1	KJ194784	Romania	2012
F1	KJ194787	Romania	2012
F1	KJ194793	Romania	2012
F1	KJ194795	Romania	2011
F1	KJ194800	Romania	2012
F1	KJ194807	Romania	2013
F1	KJ194817	Romania	2013
F1	KJ194819	Romania	2013
F1	KJ194821	Romania	2013
F1	KJ194824	Romania	2013
F1	KJ194827	Romania	2013
F1	KJ194829	Romania	2013
F1	KJ194830	Romania	2013
F1	KJ194831	Romania	2013
F1	KJ395655	Cape Green	2010
F1	KJ395661	Cape Green	2010
F1	KJ395663	Cape Green	2011
F1	KJ395695	Cape Green	2011
F1	KJ395718	Cape Green	2011

Source subtype	Accession number or id	Country	Sample year
F1	KJ395720	Cape Green	2011
F1	KJ395723	Cape Green	2011
F1	KJ395725	Cape Green	2011
F1	KJ636022	Cyprus	2012
F1	KJ636027	Cyprus	2012
F1	KJ658999	Brazil	2012
F1	KJ659002	Brazil	2012
F1	KJ849782	Brazil	2010
F1	KJ849791	Brazil	2010
F1	KJ883088	Spain	2011
F1	KJ883097	Spain	2011
F1	KJ883116	Spain	2012
F1	KJ883129	Spain	2013
F1	KM283977	Poland	2013
F1	KM851047	Brazil	2012
F1	KM851066	Brazil	2012
F1	KM851076	Brazil	2012
F1	KM851133	Brazil	2012
F1	KM851134	Brazil	2012
F1	KM851146	Brazil	2012
F1	KM851157	Brazil	2012
F1	KM851167	Brazil	2012
F1	KP121018	Turkey	2012
F1	KP121143	Turkey	2014
F1	KP121154	Turkey	2014
F1	KP231376	Romania	2013
F1	KP231377	Romania	2013
F1	KP231390	Romania	2013
F1	KP231396	Romania	2013
F1	KP231404	Romania	2011
F1	KP688144	Cuba	2008
BF1	KR066125	Brazil	2013
BF1	KR066226	Brazil	2013
F1	KR066271	Brazil	2013
F1	KR066279	Brazil	2011
F1	KT203239	Brazil	2015
F1	KT203240	Brazil	2015
F1	KT203242	Brazil	2015
F1	KT203257	Brazil	2015
BF1	KT355892	Brazil	2009
BF1	KT355895	Brazil	2009
BF1	KT355897	Brazil	2009
F1	KT355899	Brazil	2009
F1	KT355914	Brazil	2009
BF1	KT355918	Brazil	2009

Source subtype	Accession number or id	Country	Sample year
BF1	KT355942	Brazil	2009
BF1	KT355944	Brazil	2009
BF1	KT355949	Brazil	2009
F1	KT355973	Brazil	2009
F1	KT355985	Brazil	2009
F1	KT355987	Brazil	2009
F1	KT378870	China	2009
F1	KT427663	Brazil	2010
F1	KT427774	Brazil	2010
F1	KT737285	Brazil	2014
F1	KT737289	Brazil	2014
F1	KT737290	Brazil	2014
F1	KT737306	Brazil	2014
F1	KT737327	Brazil	2014
F1	KT737328	Brazil	2014
F1	KT737342	Brazil	2014
F1	KT737352	Brazil	2014
F1	KT737353	Brazil	2014
F1	KT737360	Brazil	2014
F1	KT743138	Brazil	2007
F1	KT747516	Brazil	2007
F1	KT833433	Brazil	2009
F1	KT833439	Brazil	2008
F1	KT833521	Brazil	2008
F1	KT950973	Brazil	2012
F1	KT950985	Brazil	2012
F1	KT950991	Brazil	2012
F1	KT950997	Brazil	2012
F1	KT951004	Brazil	2012
F1	KT951010	Brazil	2012
F1	KT951012	Brazil	2012
F1	KT951014	Brazil	2012
F1	KT951024	Brazil	2012
F1	KT951031	Brazil	2012
F1	KT951035	Brazil	2012
F1	KT951048	Brazil	2012
F1	KT951057	Brazil	2012
F1	KT998121	French Guiana	2009
F1	KU052741	French Guiana	2006
F1	KU168274	Romania	2003
F1	KU168276	France	2004
F1	KU685566	Spain	2015
F1	KU749395	Brazil	2010
F1	KX139355	Nigeria	2012
F1	KX158998	Romania	2013

Source subtype	Accession number or id	Country	Sample year
F1	KX159015	Romania	2013
F1	KX159050	Romania	2014
F1	KX159053	Romania	2014
F1	KX159062	Romania	2014
F1	KX159067	Romania	2014
F1	KX357219	Brazil	2012
F1	KX357233	Brazil	2012
F1	KX357259	Brazil	2013
F1	KX357260	Brazil	2013
F1	KX357282	Brazil	2013
F1	KX357294	Brazil	2015
F1	KX357303	Brazil	2015
F1	KX534326	Spain	2016
F1	KX648645	Zimbabwe	2014
F1	KX759513	United Kingdom	2013
BF1	KX838562	Brazil	2015
BF1	KX838569	Brazil	2015
F1	KX838629	Brazil	2014
F1	KX838641	Brazil	2014
F1	KX838732	Brazil	2014
F1	KX838760	Brazil	2015
F1	KX838771	Brazil	2015
F1	KX838815	Brazil	2015
BF1	KX838818	Brazil	2015
F1	KX838821	Brazil	2015
F1	KX838853	Brazil	2015
F1	KX887680	Brazil	2015
F1	KX887694	Brazil	2015
F1	KX887695	Brazil	2015
F1	KX887782	Brazil	2015
F1	KX887815	Brazil	2015
F1	KX887919	Brazil	2015
F1	KX887923	Brazil	2015
F1	KX887965	Brazil	2015
F1	KX887969	Brazil	2015
F1	KX887983	Brazil	2015
F1	KX888207	Brazil	2015
F1	KX888304	Brazil	2015
F1	KX888309	Brazil	2015
F1	KX888391	Brazil	2015
F1	KX888945	Brazil	2015
F1	KY386715	Portugal	2013
BF1	KY549858	Brazil	2013
BF1	KY549859	Brazil	2013
F1	KY581394	Brazil	2014

Source subtype	Accession number or id	Country	Sample year
F1	KY581423	Brazil	2014
F1	KY581427	Brazil	2014
F1	KY989955	Spain	2015
F1	LC170741	Japan	2013
F1	LT578264	Central African Republic	2009
F1	MF109434	United Kingdom	2014
F1	MF109661	United Kingdom	2013
F1	MF157738	Spain	2006
F1	MF403297	Spain	2013
F1	MF403298	Spain	2012
F1	MF403299	Spain	2012
F1	MF403300	Spain	2013
F1	MF403301	Spain	2007
F1	MF403302	Spain	2012
F1	MF403305	Spain	2013
F1	MF403307	Spain	2010
F1	MF403309	Spain	2013
F1	MF403310	Spain	2007
F1	MF403311	Spain	2013
F1	MF403312	Spain	2011
F1	MF403313	Spain	2013
F1	MF403317	Spain	2012
F1	MF403318	Spain	2013
F1	MF403319	Spain	2013
F1	MF403320	Spain	2009
F1	MF403322	Spain	2013
F1	MF403323	Spain	2010
F1	MF403324	Spain	2012
F1	MF573259	Brazil	2012
F1	MF594878	Brazil	2013
F1	MG365762	Brazil	2012
F1	MG365763	Brazil	2001
F1	MG365764	Brazil	2012
F1	MG365766	Brazil	2011
F1	MG365768	Brazil	2011
F1	MG365854	Brazil	2012
BF1	MG365855	Brazil	2012
F1	MG365857	Brazil	2012
BF1	MG365863	Brazil	2011
BF1	MG365869	Brazil	2009
F1	MG365872	Brazil	2008
BF1	MG365873	Brazil	2008
F1	MG365874	Brazil	2008
F1	MG941988	United States	2011
F1	MG942455	United States	2012

Source subtype	Accession number or id	Country	Sample year
F1	MG943024	United States	2014
F1	MG943639	United States	2016
F1	MH471670	Germany	2013
F1	MH471826	Germany	2013
F1	MH471851	Germany	2013
F1	MH472157	Germany	2015
F1	MH472209	Germany	2013
F1	MH472316	Germany	2016
F1	MH472508	Germany	2017
F1	MH472512	Germany	2017
F1	MH663718	United Kingdom	2012
F1	MH663751	United Kingdom	2014
F1	MH666975	Russian Federation	2015
F1	MH746263	Bulgaria	2008
F1	MK177663	Spain	2014
F1	MK177687	Spain	2015
F1	MK177701	Spain	2018
F1	MK177758	Spain	2017
F1	MK177764	Spain	2018
F1	MK177767	Spain	2018
F1	MK177773	Spain	2015
F1	MK543515	Angola	2018
F1	MK543516	Angola	2018
F1	MK543522	Angola	2018
F1	MK543538	Angola	2018
F1	MK543544	Angola	2018
F1	MK720299	Cameroon	2018
F1	MN014521	Spain	2014
F1	MN014549	Spain	2014
F1	MN014554	Spain	2015
F1	MN133138	Italy	2013
F1	MN133179	Italy	2014
F1	MN178953	Democratic Republic of Congo	2008
F1	MN178975	Democratic Republic of Congo	2008
F1	MN179001	Democratic Republic of Congo	2008
F1	MN179059	Democratic Republic of Congo	2008
F1	MN179116	Democratic Republic of Congo	2008
F1	MN179191	Democratic Republic of Congo	2008
F1	MN485989	Belgium	2016
F1	MN486030	Belgium	2017
F1	MN908888	China	2010
F1	MN908890	China	2012
F1	MT222953	United Kingdom	2015

TABLE II
Identification of the analysed sequences of NFLG53 dataset

Source subtype	Accession number	Country	Sample year
F1	AB485656	Brazil	1990
F1	AB485657	Brazil	1990
F1	AB485658	Romania	1996
F1	AF005494	Brazil	1993
F1	AF075703	Finland	1993
F1	AF077336	Belgium	1993
F1	AJ249238	France	1996
F1	AY173957	Brazil	1989
F1	AY173958	Brazil	1989
F1	DQ189088	Argentina	2002
F1	DQ979023	Spain	2006
F1	DQ979024	Spain	2006
F1	DQ979025	Spain	2006
F1	EU446022	Germany	2008
F1	FJ670516	Spain	2002
F1	FJ771006	Brazil	2002
F1	FJ771008	Brazil	2006
F1	FJ771009	Brazil	2006
F1	FJ771010	Brazil	2007
F1	FJ900266	Angola	2006
F1	FJ900267	Angola	2006
F1	FJ900268	Angola	2006
F1	GQ290462	Russia	2008
F1	JF683771	Cyprus	2008
F1	JX140671	Spain	2011
F1	KJ849782	Brazil	2010
F1	KJ849791	Brazil	2010
F1	KJ883138	Spain	2011
F1	KJ883142	Spain	2010
F1	KJ883149	Spain	2011
F1	KJ883152	Spain	2012
F1	KT276260	Spain	2014
F1	KT427774	Brazil	2010
F1	KT427814	Brazil	2010
F1	KU168274	Romania	2003
F1	KU168276	France	2004
F1	KU749395	Brazil	2010
F1	KU749396	Brazil	2011
F1	KY514084	Spain	2014
F1	KY639272	Spain	2011
F1	KY989955	Spain	2015

Source subtype	Accession number	Country	Sample year
F1	MF109434	United Kingdom	2014
F1	MF109526	United Kingdom	2014
F1	MF109640	United Kingdom	2013
F1	MG365762	Brazil	2012
F1	MG365763	Brazil	2001
F1	MG365764	Brazil	2012
F1	MG365766	Brazil	2011
F1	MG365767	Brazil	2008
F1	MG365768	Brazil	2011
BF1	MG365770	Brazil	2002
BF1	MG365771	Brazil	2001
F1	MK041565	Brazil	2016
